# Use of new approach methodologies (NAMs) to meet regulatory requirements for the assessment of tobacco and other nicotine-containing products

**DOI:** 10.3389/ftox.2022.943358

**Published:** 2022-09-09

**Authors:** Jacqueline Miller-Holt, Holger P. Behrsing, Amy J. Clippinger, Carole Hirn, Todd J. Stedeford, Andreas O. Stucki

**Affiliations:** ^1^ Scientific and Regulatory Affairs, JT International SA, Geneva, Switzerland; ^2^ Institute for in Vitro Sciences, Inc., Gaithersburg, MD, United States; ^3^ PETA Science Consortium International e V, Stuttgart, Germany; ^4^ Independent Consultant, Washington, DC, United States

**Keywords:** tobacco and tobacco product, nicotine-containing products, new approach methodologies (NAMs), regulatory flexibility, toxicity testing, *in vitro*, *in silico*

## Abstract

Regulatory frameworks on tobacco and other nicotine-containing products (TNCP) continue to evolve as novel products emerge, including electronic nicotine delivery systems (e.g., electronic cigarettes or vaping products), heated tobacco products, or certain smokeless products (e.g., nicotine pouches). This article focuses on selected regulations for TNCPs that do not make health claims, and on the opportunities to use new approach methodologies (NAMs) to meet regulatory requirements for toxicological information. The manuscript presents a brief overview of regulations and examples of feedback from regulatory agencies whilst highlighting NAMs that have been successfully applied, or could be used, in a regulatory setting, either as stand-alone methods or as part of a weight-of-evidence approach to address selected endpoints. The authors highlight the need for agencies and stakeholders to collaborate and communicate on the development and application of NAMs to address specific regulatory toxicological endpoints. Collaboration across sectors and geographies will facilitate harmonized use of robust testing approaches to evaluate TNCPs without animal testing.

## 1 Introduction

The health risks from combustible tobacco products (e.g., cigarettes) have been known for decades. In the 20th century, converging lines of evidence were available that began to shed light on the increasing incidence of lung cancers amongst smokers around the world. These evidence streams included population studies, studies in experimental animals, cellular pathology, and the identification of cancer-causing chemicals in cigarette smoke (Proctor, 2012).

**FIGURE 1:**
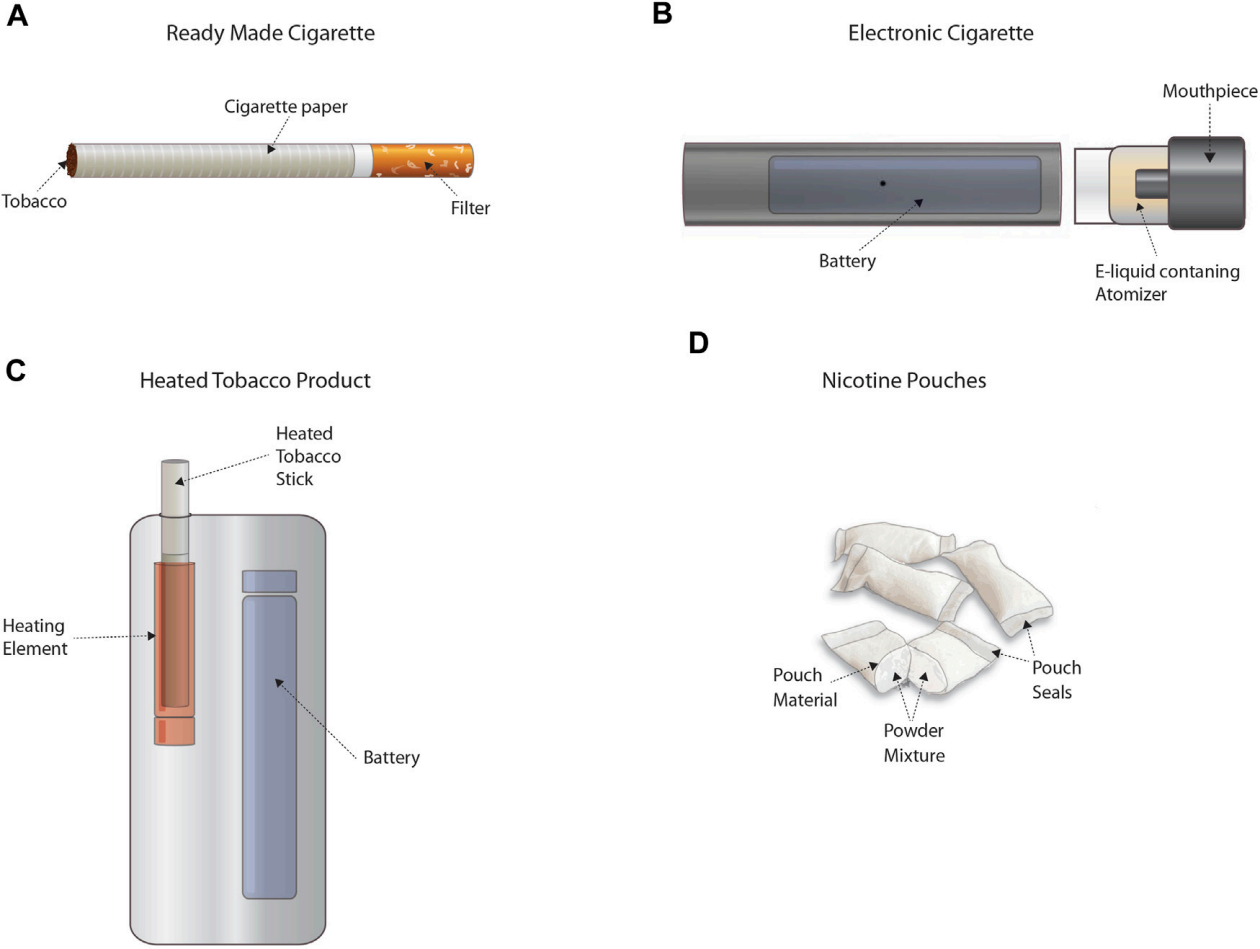
Representative tobacco and other nicotine-containing products (TNCP). Panel **(A)** provides an example of a ready-made cigarette that delivers nicotine in smoke formed through combustion. Panel **(B)** provides an example of an electronic cigarette that delivers nicotine in a heated vapor/aerosol. Panel **(C)** provides an example of a heated tobacco product that delivers nicotine in a heated vapor/aerosol. Panel **(D)** provides an example of oral nicotine pouches that deliver nicotine from a powder mixture. TNCPs are defined differently throughout the various jurisdictions and may or may not include other products. Please consult the regulations of the respective geographical area for the exact definitions.

Decades later, cigarette smoking remains one of the leading preventable causes of morbidity and mortality. Today, novel tobacco and other nicotine-containing products (TNCP)—including electronic nicotine delivery systems (ENDS) (e.g., electronic cigarettes or vaping products), heated tobacco products, or certain smokeless products (e.g., nicotine pouches) (Figure 1)^1^—are available that may facilitate nicotine addicted smokers to transition to potentially lower risk alternatives and/or cessation. Regulatory frameworks around the world have mechanisms for evaluating novel TNCPs but unlike combustible cigarettes, the available data on these technologies are comparatively limited. Indeed, substantial *in vitro* and *in vivo* data have been generated for combusted tobacco products using a variety of assays to address a number of toxicological endpoints. An overview of studies previously published or provided to authorities is documented in a recent report from the German Institute for Standardisation (DIN, 2021). As regulators provide feedback to industry or as guidelines change and methodologies develop, it is clear that data from multiple assays considered in a weight-of-evidence approach will be useful to fully address the broad spectrum of health effects normally attributed to tobacco smoke. This creates challenges for developers, as well as for regulators who are tasked with determining whether these technologies may advance the decline in smoking prevalence and population harm, without unintended side effects.

In 2007, the United States (US) National Research Council (NRC, 2007) issued its groundbreaking report titled “Toxicity Testing in the 21st Century—A Vision and a Strategy”. Since this time, scientists across government, academia, non-governmental organizations, and regulated sectors have been diligently researching and applying novel testing strategies, commonly known as New Approach Methodologies (NAMs). NAMs are any technology, methodology, approach, or combination that can provide information on chemical hazard and risk assessment that replaces or reduces the use of animals, including *in silico*, *in chemico*, *in vitro*, and *ex vivo* approaches (ECHA, 2016; EPA, 2018b). NAMs are not necessarily newly developed methods, rather, it is their application to regulatory decision making or replacement of a conventional testing requirement that is new. They may be based on human cells or cell components that avoid the use of vertebrate animals and the uncertainties associated with extrapolating findings from experimental animals to humans. Therefore, NAMs may offer a unique and more efficient means of informing key events from adverse outcome pathways and exploring potential toxicological effects during the research and development (R&D) process and in regulatory filings (Peitsch et al., 2018; Luettich et al., 2021).

With the growth and increasing variants of non-combustible TNCPs, regulatory requirements have changed or are under development in several nations. In many countries, the provision of toxicological information is obligatory and can vary between specific submissions in the case of substantive product changes or for new product launches (e.g., US) and/or an annual submission for all products (e.g., Canada and the EU).

This article focuses on selected legal and regulatory frameworks for nicotine control in the US, Canada, and the European Union (EU) and the guidance, if available, for using data from NAMs in lieu of data from animals. This perspective provides select examples of opportunities where data from NAMs may be used to evaluate R&D products, as well as to inform data requirements amongst the required toxicological endpoints in certain regulatory frameworks for TNCPs. The authors acknowledge that other countries (e.g., Japan and South Korea) and organizations (e.g., World Health Organisation) are also very active in this area and have published several reports (WHO, 2021). However, this perspective focuses on information available in the US, Canada, and the EU that highlight the need for alignment throughout the world with regard to regulatory acceptance and use of NAMs.

## 2 Overview of regulatory status

### 2.1 United States

In 2009, President Barack Obama signed into law the Family Smoking Prevention and Tobacco Control Act (Tobacco Control Act; Pub.L. 111–31), giving the US Food and Drug Administration (FDA) authority to regulate TNCPs under the Federal Food Drug and Cosmetic Act (FD&C Act, 21 USC §301 et seq.; Subchapter IX, 21 USC §387). As a result, the Center for Tobacco Products (CTP) was created within FDA and requires CTP to review and approve or deny new TNCPs before market entry.

For any new TNCP, under FD&C Section 910, an application is mandated, along with the requirement to obtain an FDA marketing authorization order. Marketing authorization may be acquired using one of the following pathways (21 USC §387j):

• Substantial equivalence (SE) report for new TNCPs that have the same characteristics as another product with market approval or its exemption when applicable
• Premarket Tobacco Product Application (PMTA)

The information to be included into SE reports is judged on a case-by-case basis, primarily depending on the changes proposed to the new product compared to a predicate product in a report. The current regulatory impact analysis on SE reports indicates that whatever data are submitted, it should include test protocols, quantitative acceptance criteria, and test results as well as clearly identify when national or international standards are used to test the new and predicate products in addition to explaining any deviations from the standard or stating if no testing standards were used. In this respect, the guidelines are sufficiently broad to allow the submission of information from NAMs (FDA, 2021a).

In the guidance for industry on PMTAs for ENDS, CTP documented that a data package might require the following, non-clinical investigations, if available (FDA, 2019a):

• *In vitro* toxicology studies (e.g., genotoxicity studies or cytotoxicity studies)
• Computational modeling of the toxicants in the product (to estimate the toxicity of the product)
• *In vivo* toxicology studies (to address unique toxicology issues that cannot be addressed by alternative approaches)

Throughout this guidance document, CTP states its support for reducing, replacing, and/or refining the use of animal testing where adequate and scientifically valid NAMs can be substituted (FDA, 2019a). CTP encourages sponsors to meet with them early in the development process to discuss the suitability and acceptability of NAMs for informing potential hazards from the new TNCP. Furthermore, the finalized 2019 guidance document states “[i]n the absence of toxicological data for a particular toxicant of concern, we recommend that you consider computational modeling using surrogate chemical structures” (FDA, 2019a). The guidance goes on to state “[i]f you plan to conduct any computational modeling, we suggest that you meet with CTP to specifically address this issue”.

In its final rule, CTP provides information on the types of investigations that applicants must submit as part of a PMTA, if published, known to, or which should reasonably be known to an applicant including, but not limited to, genotoxicity, carcinogenicity, respiratory toxicity, cardiac toxicity, reproductive and developmental toxicity, and chronic (repeated dose) toxicity of the new TNCP relative to other TNCPs. It includes human exposure studies, *in silico* computational toxicology techniques, risk assessments, *in vitro* toxicology studies, published reports of *in vivo* toxicology studies, and, if necessary, new *in vivo* toxicology studies. Additionally, CTP reconfirms in the final rule its support of reducing reliance on animal testing where adequate and scientifically valid NAMs can be substituted (FDA, 2021b). While the final rule and guidance for industry suggest that NAMs are generally deemed acceptable, CTP did not provide detailed information on specific NAMs and the extent to which they can be used.

CTP’s flexibility with considering the use of NAMs is representative of broader initiatives at FDA that have been ongoing for more than a decade. For example, in its 2011 strategic plan titled “Advancing Regulatory Science at FDA,” FDA reiterated its general support for the use of NAMs where transformation of toxicology was identified as a key scientific priority that offers enormous opportunities (FDA, 2011). In its 2017 Predictive Toxicology Roadmap, which was published to guide FDA’s six product centers—including CTP—in the development and use of new technologies, FDA reiterated its support for the use of NAMs (FDA, 2017). More recently, FDA updated the strategic plan with a report on focus areas of regulatory sciences in which they identified that NAMs will likely provide enhanced prediction of the risk and/or safety outcomes (FDA, 2021c). The FDA is also included in the Interagency Coordinating Committee on the Validation of Alternative Methods which published a strategic roadmap in 2018 to serve as a guide for agencies and stakeholders seeking to adopt NAMs for chemical safety and risk assessments (ICCVAM, 2018).

### 2.2 Canada

The Tobacco and Vaping Products Act (TVPA) regulates the manufacture, sale, labelling, and promotion of TNCPs (S.C. 1997, c.13). TVPA stipulates that Canada’s Governor in Council may make additional regulations prescribing information that manufacturers must submit to the Minister about R&D related to TNCPs and their emissions, including health effects and hazardous properties.

The Tobacco Reporting Regulations (TRR; Statutory Orders, and Regulations [SOR]/2000–273, CDC, 2000) requires an annual report be generated on the toxicity of cigarette emissions. The Regulations refer to the following *in vitro* toxicity tests for Mainstream Tobacco Smoke: Bacterial Reverse Mutation, Neutral Red Uptake Assay, and *In Vitro* Micronucleus Assay, and an annual report on R&D activities as indicated in Section 15 of the TRR in addition to sales, emissions, and contents of the product.

The TRR currently includes testing requirements for tobacco smoke (i.e., combustible tobacco products), but not other nicotine-containing products. The amendment of the TRR as well as implementing vaping reporting regulations were initiated in 2017 and are part of Health Canada’s Forward Regulatory Plan 2021–2023 and 2022–2024 but have not been published yet (HC, 2021, 2022).

### 2.3 European Union

In the EU, the Tobacco Products Directive (TPD; Directive 2014/40/EU, 2014) sets the minimum requirements (e.g., health warnings and reporting) for TNCPs to be placed on the market in EU, 2015a Member States.

For tobacco products, Article 5 (3) requires the submission of a list of ingredients with relevant toxicological data for the ingredients in burnt and unburnt form, as appropriate. In particular, any effects on human health or addictiveness of the ingredients shall be reported. Similar information is required for ENDS, per Article 20. For novel TNCPs, Article 19 requires the submission of any available scientific studies on toxicity, addictiveness, and attractiveness of the novel TNCP. Additionally, Article 6 (2) requires manufacturers and importers of cigarettes and Roll-Your-Own tobacco that contain an additive from the priority list established by Commission Implementing Decision (EU, 2015b) 2016/787 to carry out comprehensive studies (EU, 2016).

The field descriptors for toxicological data submission are documented in Commission Implementing Decision 2015/2186 for tobacco products and 2015/2183 for electronic cigarettes.

The toxicological data reporting indicates that any type of study may be submitted, implying the possibility to submit NAMs for informing potential hazard concerns for genotoxicity, carcinogenicity, respiratory toxicity, reproductive toxicity, cardiopulmonary toxicity, and any other type of toxicity. Several EU Member States, such as Belgium, Estonia, Germany, and Slovakia, have banned the use of animals for the development and testing of tobacco products.

Individual Member States “may also require manufacturers or importers to carry out studies as may be prescribed by the competent authorities in order to assess the effects of ingredients on health, taking into account, inter alia, their addictiveness and toxicity”. Member States also require any new or updated information to be submitted to their competent authorities or may require additional tests or information for novel TNCPs.

In the recently published “Support study to the report on the application of Directive 2014/40/EU”, Member State feedback indicated that there was a “lack of guidance on how e.g. toxicological studies should be assessed” (EC, 2021). It was also implied that there was a range of scientific approaches submitted by different manufacturers and, as such, when submitting information from NAMs, it is recommended that companies provide clear guidance on how to interpret the data.

## 3 Application of NAMs

For the *in vitro* evaluation of inhaled TNCP, aerosols or smoke are generated and particulates are captured on a filter pad (total particulate matter, TPM), and the remaining gas-vapor phase (GVP) bubbled through a liquid or the cells are directly exposed at an air-liquid interface. Examples of methodologies can be found in the references from Table 1 (e.g., Moore et al., 2020; Smart and Phillips, 2021; Bishop et al., 2020).

**TABLE 1:** Examples of available NAMs currently applied in regulatory settings and NAMs under development or other resources, that could be used to develop Integrated Approaches to Testing and Assessment (IATA) for tobacco and other nicotine containing products. This table features only select examples and serves as conversation starter. Mentions of specific NAMs are neither an endorsement of this particular method nor does it mean that no other NAMs exist to fulfil the information requirements for this particular endpoint.

Endpoint	NAM/Subject matter	Regulatory relevance, recognition or potential application
Examples of NAMs currently or soon expected to be applied in a regulatory setting		
Dermal Toxicity (topical)	Defined Approaches on Skin Sensitisation (DASS)	OECD MAD (TG 497, 442C, 442D, 442 E); EPA TSCA List of Alternative Test Methods and Strategies (or New Approach Methodologies), —The List; EPA OCSPP Skin Sensitization Policy
	DASS automated workflow (AW)/OECD QSAR Toolbox 4.5	
	Derek Nexus v.6.1.0/Derek KB 2020 1.0	
	*In Chemico* Skin Sensitisation—Assays Addressing the AOP Key Event on Covalent Binding to Proteins (Direct Peptide Reactivity Assay and Amino Acid Derivative Reactivity Assay)	
	*In Vitro* Skin Sensitisation—Assays Addressing the AOP Key Event on Keratinocyte Activation (KeratinoSens™ and LuSens)	
	*In Vitro* Skin Sensitisation—Assays Addressing the AOP Key Event on Activation of Dendritic Cells (h-CLAT, U-SENS^TM^, and IL-8 Luc assays)	
	*In Vitro* Skin Irritation—Reconstructed Human Epidermis Test Method	OECD MAD (TG 439, 431, 435; GD No. 203);; EPA TSCA List of Alternative Test Methods and Strategies (or New Approach Methodologies), —The List
	*In Vitro* Skin Corrosion—Reconstructed Human Epidermis Test Method	
	*In Vitro* Membrane Barrier Test Method for Skin Corrosion (Corrositex)	
Carcinogenicity	OncoLogic™ (version 8.0) (fibers, metals, polymers)	EPA TSCA List of Alternative Test Methods and Strategies (or New Approach Methodologies), —The List
	OncoLogic™ (version 9.0) (organic chemicals)	
	*In Vitro* Cell Transformation Assays (non-genotoxic carcinogens)	EURL ECVAM TM 2004–07 (EU); OECD GD No. 214 & 231; EPA TSCA List of Alternative Test Methods and Strategies (or New Approach Methodologies), —The List
Mutagenicity	Bacterial Reverse Mutation Test (Ames Test) (	OECD MAD (TG 471, 473, 476, 487, 490); EPA TSCA List of Alternative Test Methods and Strategies (or New Approach Methodologies), —The List
	*In Vitro* Mammalian Chromosomal Aberration Test	
	*In Vitro* Mammalian Cell Gene Mutation Tests using the Hprt and xprt Genes	
	*In Vitro* Mammalian Cell Micronucleus Test	
	*In Vitro* Mammalian Cell Gene Mutation Tests using the Thymidine Kinase Gene	
	Validation of the 3D reconstructed human skin Comet assay, an animal-free alternative for following-up positive results from standard *in vitro* genotoxicity assays (Pfuhler et al., 2021a)	Accepted into the OECD, 2022 test guideline development program (OECD, 2021)
	Validation of the 3D reconstructed human skin micronucleus (RSMN) assay: an animal-free alternative for following-up positive results from standard *in vitro* genotoxicity assays (Pfuhler et al., 2021b)	
Pulmonary Toxicity	Reconstructed airway epithelium (MucilAir) evaluated using multiple endpoints for acute irritation (EPA, 2018a; McGee Hargrove et al., 2021)	Applied to EPA FIFRA SAP 2018; EPA 2021, Draft Risk Assessment 2021; EPA TSCA List of Alternative Test Methods and Strategies (or New Approach Methodologies), Appendix B—Other Information or Strategies
	Computational fluid dynamics for exposure assessment combined with reconstituted airway epithelium (MucilAir) (Corley et al., 2021; McGee Hargrove et al., 2021)	
	A weight-of-the-evidence (WoE) approach for evaluating, in lieu of animal studies, the potential of a novel polysaccharide polymer to produce lung overload (Ladics et al., 2021)	Was used by EPA in a WoE approach to revoke a significant new use rule (SNUR)
Examples of work supporting NAMs expansion into additional organs and endpoints		
Cardiotoxicity	PBK based NAM for the prediction of cardiotoxicity (Shi et al., 2021)	May be used as part of WoE approach
	Cardio quickPredict (metabolites-based assay utilizing human induced pluripotent stem cells) (Simms et al., 2022)	
	PBK model-guided evaluation of methadone on human-induced pluripotent stem cell-derived cardiomyocytes, comparison to *in vivo* data (Shi et al., 2020)	
Cardiovascular toxicity	INSPIRE: A European training network to foster research and training in cardiovascular safety pharmacology (Guns et al., 2020)	Overview of cardiovascular toxicity testing (not specifically NAMs)
Reproductive/developmental toxicity	devTOX quickPredict (metabolomics biomarker-based assay that utilizes human induced pluripotent stem cells) (Simms et al., 2020)	May be used as part of a WoE approach
	Rethinking Developmental Toxicity Testing (Scialli et al., 2018)	NAM Applicability Reviews
	Beyond AOPs: A mechanistic evaluation of NAMs in DART Testing (Rajagopal et al., 2022)	
Pulmonary Toxicity	Multi-path Particle Dosimetry (https://www.ara.com/mppd/)	May be used as part of an assessment instead of computational fluid dynamics
	Mucociliary Clearance (ciliary beat frequency) (Luettich et al., 2021)	May be used to develop NAMs specific to this human adverse outcome relevant for TNCPs
	Human air-liquid-interface organotypic airway tissue models derived from primary tracheobronchial epithelial cells-overview and perspectives (Cao et al., 2021)	3D Test System review that may be used to develop NAMs
	Eurofins SafetyScreen44 and BioMap Diversity 8 Panel; ToxCast data, an *in vitro* cell stress panel and high-throughput transcriptomics; *in silico* alerts for genotoxicity were followed up with the ToxTracker tool (Baltazar et al., 2020)	May be used as part of a WoE approach
	*In vitro* alveolar macrophage assay for predicting the short-term inhalation toxicity of nanomaterials (Wiemann et al., 2016)	
Multi-endpoint/Approach-based	An FDA/CDER perspective on nonclinical testing strategies: Classical toxicology approaches and NAMs (Avila et al., 2020)	Review of NAMs and target organ toxicity by FDA/CDER
	Assessment of *in vitro* COPD models for tobacco regulatory science (Behrsing et al., 2016)	Best Practices Recommendations
	*In vitro* exposure systems and dosimetry assessment for NAMs (Behrsing et al., 2017)	
	Recommendations for the optimal generation and use of *in vitro* assay data for tobacco product evaluation (Moore et al., 2020)	

The below examples illustrate the current challenges in the application of NAMs for the risk assessment of TNCPs. On the one hand, manufacturers are encouraged to use and submit data from NAMs and not to conduct animal testing for these products (example 3.1). On the other hand, regulatory agencies seem to struggle to interpret some of the data generated with NAMs for risk assessment purposes. As described in Section 2, the guidance documents are unclear in regards to NAMs that are currently readily used and accepted (as in example 3.2) and which NAMs may require the generation of additional information to fulfil the requirements for regulatory acceptance (as in example 3.3). Ambiguous guidance further leads to increased testing on animals because applicants tend to submit more information if the requirements are unclear (example 3.3). This illustrates the need for industry and regulatory agencies to collaborate and develop Integrated Approaches to Testing and Assessment (IATA) without the use of animals for TNCPs. Table 1 lists examples of available NAMs that could be included in a regulatory submission, as well as NAMs under development that could be incorporated as part of a regulatory submission package.

### 3.1 Example highlighting acknowledgement of the need to accept NAMs

In December 2020, a Joint Action for Tobacco Control (JATC) review panel set-up to aid EU Member States in the evaluation of data submitted for priority additives issued a report. A recurring comment throughout the report was that “the *in vitro* tests included in the newly performed industry studies are not sufficient to perform an evaluation of the CMR (carcinogenic, mutagenic or reprotoxic) properties, since *in vivo* studies are required to address this issue. Nevertheless, the review panel acknowledges that new *in vivo* studies regarding tobacco products are neither appropriate nor allowed for ethical reasons”. The panel went on to comment “we do not have a proposed scientific methodology for fulfilling our request for evidence...” and indicated that if there is a revision of the TPD “the possible use of some assessment methodologies (e.g. Mode of Action and Adverse Outcome Pathway), which do not necessarily need new animal studies, should be considered” (JATC, 2020).

### 3.2 Example demonstrating how NAMs can be used to inform regulatory decision making

In the decision summary of a PMTA, the CTP Technical Project Lead (TPL) stated “Results from the *in vitro* toxicology studies demonstrated that combusted cigarette smoke fractions (total particulate matter (TPM), gas vapor phase (GVP), or both) were mutagenic, cytotoxic, and genotoxic. By contrast, even at the maximum dose levels tested, neither the TPM nor GVP from any of the aerosols of all the new products or ENDS market comparisons was mutagenic, cytotoxic, or genotoxic under the test conditions.” (FDA, 2019b).

### 3.3 Example reiterating the importance of involving the regulatory agency early in the experimental development process and to clearly describe the NAMs

In the decision summary of a PMTA where five separate *in vitro* organotypic studies were submitted, it was noted “The experimental approach taken in these studies included using methods that are exploratory, have not been independently validated, and have unknown utility for regulatory use. The applicant attempts to extrapolate from acute exposure studies with naïve tissues that have little or no genetic variability to predict toxicity in a diverse population with a history of cigarette smoking. This limits the use of these data.” In the same decision summary, a nicotine pharmacokinetic (PK) study with rats was dismissed stating “[t]his study does not provide relevant information for determining the health effects of [the product]; however, human PK studies were submitted and are more informative” (FDA, 2019c).

## 4 Discussion and next steps

For ethical and scientific reasons, there is a need to use reproducible and human-relevant testing approaches without the use of animals to better understand the potential adverse effects of TNCPs on humans. Investments have been made in the development of NAMs for assessing endpoints of relevance to TNCPs, as well as in the development of adverse outcome pathways that demonstrate the biological relevance of the NAMs. Human relevant NAMs have the potential to efficiently determine whether pre-market technologies should be abandoned due to specific hazard concerns, thereby avoiding unintended human health hazards that may only be identified during post-market surveillance. In addition to their use in product development, fit-for-purpose NAMs may be used to generate human relevant data that fulfill regulatory data requirements.

Section 2 highlighted the differences in the current regulatory frameworks of three exemplary regions and shows the need for more aligned regulations and clear guidance for the use of NAMs. In addition, to facilitate regulatory acceptance, applicants should clearly describe their NAM, including the context in which the NAM will be used (e.g., for screening/prioritization or quantitative risk assessment), the relevance of the model system to human biology and mechanisms of toxicity, and how the NAM informs the toxicological issue/gap. These are important aspects to communicate to regulators because the use of NAM-based data may not provide information that is typically used in regulatory risk assessments, e.g., no-observed-adverse-effect concentration. Rather, the NAM-based data may inform whether a particular substance should be included amongst a category of chemical substances with known hazard concerns or the data may inform a specific key event in an adverse outcome pathway.

It is also important to keep in mind that a NAM may generate data that are different than data generated from vertebrate animals but are more relevant and mechanistically informative for predicting potential hazards to humans. A representative example in the context of inhalation toxicology is that rodents are obligate nose breathers, whereas humans are oronasal breathers and primarily mouth breathers when using inhalable products (e.g., cigarettes). Species-specific differences in dosimetry of substances can be accounted for using currently available NAMs [e.g., Multiple-Path Particle Dosimetry (MPPD)], which may also be used to inform the most relevant regions of the respiratory tract for investigation in human cells. Especially because regulators may not be as familiar with NAM data, it is essential for the regulated community to provide scientific justification for using NAM data in lieu of vertebrate animal data in a regulatory filing.

The above considerations emphasize the need for early engagement between regulators and the regulated community when utilizing NAMs as part of regulatory filings. These interactions will allow for discussion about how NAM data can be used to answer regulatory questions and foster further optimization of testing approaches to meet regulatory needs and best predict human health effects. Ultimately, the advancement of NAMs will require engagement from both sides to ensure a mutual understanding that the proposed NAMs are providing information in the context of the current paradigm for regulatory risk assessments or explaining how the NAMs may be incorporated to inform an alternative approach for assessment. In addition, consistency and transparency in how agencies consider and use submitted data in their decision making will allow the regulated community to more rapidly meet agency expectations.

Overall, there is a need for broader collaboration between regulatory authorities across geographical areas, between regulatory authorities and their stakeholders, and across sectors (e.g., industrial chemicals, agrochemical, or cosmetics) in order to harmonize best scientific practices. To help eliminate the use of animals in the risk assessment of TNCPs, it is recommended to:

• encourage pre-submission meetings between the agency and applicants to get early feedback on proposed NAMs
• provide consistent and transparent evaluations of application dossiers
• establish an international government-to-government collaboration initiative (similar to e.g., Accelerating the Pace of Chemical Risk Assessment [APCRA] for industrial chemicals)
• establish an open exchange forum between agencies, the regulated community, and other experts to discuss NAMs and how they can be applied for risk assessment purposes
• conduct retrospective reviews of submitted applications (such as PMTAs) with an aim to identify clear information needs for risk assessments
• organize regular workshops and webinars on the use of NAMs in risk assessment
• publicly share data to avoid duplicative testing and aid in read-across (e.g., the INTERVALS database)

Collaborative efforts will advance the harmonized use and acceptance of reliable, fit-for-purpose NAMs that inform human biology. Regulatory alignment on such approaches will not only aid with the development of novel TNCPs but will also aid other industries with evaluating their chemistries and developing products that are of lower risk to human health and the environment.

## Data Availability

The original contributions presented in the study are included in the article/Supplementary Material, further inquiries can be directed to the corresponding authors.

## Footnotes

1 TNCPs are defined differently throughout the various jurisdictions and may or may not include additional products. Please consult the regulations of each geographical area for the exact definitions.
